# Supplementary material for: Resolving the full spectrum of human genome variation using Linked-Reads
Source: Genome Res. 2019 Apr;29(4):635–45. doi: 10.1101/gr.234443.118 (PMC6442396; doi:10.1101/gr.234443.118)
Supplement: Supplemental Material [file supp_gr.234443.118_Supplemental_longranger-2.2.zip › longranger-2.2.tar.bz2!/lr2.2-public/lib/stan/cmdstan/stan/doc/api/html/initialize_8hpp_source.html]

Stan: src/stan/math/matrix/initialize.hpp Source File


|  |  |
| --- | --- |
|  | Stan  2.5.0  probability, sampling & optimization |


- Main Page
- Related Pages
- Namespaces
- Classes
- Files

- File List
- File Members

All Classes Namespaces Files Functions Variables Typedefs Enumerations Enumerator Friends Macros Pages

- src
- stan
- math
- matrix

initialize.hpp

Go to the documentation of this file.

1 #ifndef STAN\_\_MATH\_\_MATRIX\_\_INITIALIZE\_HPP

2 #define STAN\_\_MATH\_\_MATRIX\_\_INITIALIZE\_HPP

3

4 #include <vector>

5 #include <boost/type\_traits/is\_arithmetic.hpp>

6 #include <boost/utility/enable\_if.hpp>

7 #include <stan/math/matrix/Eigen.hpp>

8

9 namespace stan {

10

11  namespace math {

12

13  // initializations called for local variables generate in Stan

14  // code; fills in all cells in first arg with second arg

15

16  template <typename T>

17  inline void initialize(T& x, const T& v) {

18  x = v;

19  }

20  template <typename T, typename V>

21  inline

22  typename boost::enable\_if\_c<boost::is\_arithmetic<V>::value, void>::type

23  initialize(T& x, V v) {

24  x = v;

25  }

26  template <typename T, int R, int C, typename V>

27  inline void initialize(Eigen::Matrix<T,R,C>& x, const V& v) {

28  for (int i = 0; i < x.size(); ++i)

29  initialize(x(i),v);

30  }

31  template <typename T, typename V>

32  inline void initialize(std::vector<T>& x, const V& v) {

33  for (size\_t i = 0; i < x.size(); ++i)

34  initialize(x[i],v);

35  }

36

37  }

38 }

39 #endif

stan::math::initialize

void initialize(T &x, const T &v)

**Definition:** initialize.hpp:17

Eigen.hpp


---

[ Stan Home Page ]

*© 2011–2014,
Stan Development Team.*
